# Serum Levels of Inflammatory Proteins Are Associated With Peripheral Neuropathy in a Cross-Sectional Type-1 Diabetes Cohort

**DOI:** 10.3389/fimmu.2021.654233

**Published:** 2021-03-31

**Authors:** Sharad Purohit, Paul Minh Huy Tran, Lynn Kim Hoang Tran, Khaled Bin Satter, Mingfang He, Wenbo Zhi, Shan Bai, Diane Hopkins, Melissa Gardiner, Chandramohan Wakade, Jennifer Bryant, Risa Bernard, John Morgan, Bruce Bode, John Chip Reed, Jin-Xiong She

**Affiliations:** ^1^ Center for Biotechnology and Genomic Medicine, Augusta University, Augusta, GA, United States; ^2^ Department of Obstetrics and Gynecology, Medical College of Georgia, Augusta University, Augusta, GA, United States; ^3^ Department of Undergraduate Health Professionals, College of Allied Health Sciences, Augusta University, Augusta, GA, United States; ^4^ Department of Neurology, Medical College of Georgia, Augusta University, Augusta, GA, United States; ^5^ Atlanta Diabetes Associates, Atlanta, GA, United States; ^6^ Southeastern Endocrine & Diabetes, Atlanta, GA, United States

**Keywords:** autoimmunity, chronic inflammation, type-1 diabetes, cytokines, receptors, peripheral neuropathy

## Abstract

Chronic low-grade inflammation is involved in the pathogenesis of type-1 diabetes (T1D) and its complications. In this cross-section study design, we investigated association between serum levels of soluble cytokine receptors with presence of peripheral neuropathy in 694 type-1 diabetes patients. Sex, age, blood pressure, smoking, alcohol intake, HbA1c and lipid profile, presence of DPN (peripheral and autonomic), retinopathy and nephropathy was obtained from patient’s chart. Measurement of soluble cytokine receptors, markers of systemic and vascular inflammation was done using multiplex immunoassays. Serum levels were elevated in in DPN patients, independent of gender, age and duration of diabetes. Crude odds ratios were significantly associated with presence of DPN for 15/22 proteins. The Odds ratio (OR) remained unchanged for sTNFRI (1.72, p=0.00001), sTNFRII (1.45, p=0.0027), sIL2Rα (1.40, p=0.0023), IGFBP6 (1.51, p=0.0032) and CRP (1.47, p=0.0046) after adjusting for confounding variables, HbA1C, hypertension and dyslipidemia. Further we showed risk of DPN is associated with increase in serum levels of sTNFRI (OR=11.2, p<10), sIL2Rα (8.69, p<10^-15^), sNTFRII (4.8, p<10^-8^) and MMP2 (4.5, p<10^-5^). We combined the serum concentration using ridge regression, into a composite score, which can stratify the DPN patients into low, medium and high-risk groups. Our results here show activation of inflammatory pathway in DPN patients, and could be a potential clinical tool to identify T1D patients for therapeutic intervention of anti-inflammatory therapies.

## Introduction

The morbidity and mortality associated with type-1 diabetes (T1D) is mainly related to the development of long-term micro- and macro-vascular complications (1). The microvascular damage due to hyperglycemia can lead to neuropathy, retinopathy, and nephropathy (2–4). The macro-vascular damage can lead to thrombosis (5). Neuropathies (DPN) are one of the first and more common diabetic complications and present in up to 50% of patients (6), is frequently underdiagnosed by physicians (7) and can be unrecognized by the patients (8). The major characteristic of diabetic peripheral neuropathy (DPN) is the progressive loss of nerve fibers from both the autonomic and peripheral nervous system. This loss of sensory function has major detrimental effects, including risk of foot ulcerations, amputations, and increased mortality rates (6).

The Diabetic Control and Complications Trial/Epidemiology of Diabetes and Complications (DCCT/EDIC) study suggests that despite intensive glycemic control, incidence and prevalence of DPN increased in both the subjects under intensive (7% incidence) and permissive (17% incidence) glycemic control (9, 10). The reported prevalence of diabetic peripheral neuropathy (DPN) in type 1 diabetes (T1D) patients is highly variable (11). It was 13% in a Scottish registry population study (12), 11% in the 25,000‐person T1D Exchange Clinic Network in the U.S (13) and 28% in EURODIAB IDDM Complications Study (14). Recent reports from DCCT/EDIC suggests that prevalence of DPN is 28% in T1D patients after 20 years of diabetes (9, 15, 16). In a Danish study, presence of subclinical DPN was 55.1% whereas only 2.6% individuals were confirmed to have DPN (17). The Pittsburgh Epidemiology of Diabetes Complications Study, suggested that DPN was prevalent in 18% of the 18–29-year-old individuals (18).

The high rates of DPN among youth with diabetes are a cause of concern and requires early detection and better risk factor management. Interventions to address poor glycemic control and dyslipidemia may prevent or delay debilitating neuropathic complications (17–19). The long-term complications of T1D require lifestyle changes and medications that will modify risk factors such as hyperglycemia, hyperlipidemia, and hypertension (10, 17, 18). Yet, the current age and duration of type 1 diabetes remain important predictors of developing DPN. These studies suggest some T1D patients would still develop complications despite intensive treatment. Therefore, there is a need for additional markers to identify these at-risk population.

The precise mechanisms by which DPN occurs is multi-factorial (20, 21). However, chronic low-grade inflammation is considered the root cause of development of complications in both type-1 and type-2 diabetes patients (21, 22). Previous reports have shown that serum levels of inflammatory proteins, such as TNF-α, IL-16, soluble TNF receptors (sTNFR1 and 2) were increased in the serum of T1D patients with micro-vascular (23–25) and macro-vascular complications (26). In T1D subjects with DPN, serum levels of sTNFR1 and sTNFR2 were associated with DPN after adjusting for confounders (27). The majority of the studies on inflammatory proteins in DPN is on the type-2 diabetes (T2D) patients. We have shown that serum levels of insulin like growth factor binding proteins (IGFBP), TNF-α and IL-6 pathways were able to stratify T1D patients into risk categories for a number of complications (28), including nephropathy (24) and retinopathy (25). Results presented here show that serum levels of five inflammatory proteins are associated with increased risk of DPN in T1D individuals after adjusting for age, duration, gender, hypertension and dyslipidemia. We utilized a machine learning approach to combine the serum level of several proteins into a linear predictor to stratify T1D subjects.

## Materials and Methods

### Study Population

Serum samples for this cross-sectional study were obtained from Caucasian subjects recruited into the Phenome and Genome of Diabetes Autoimmunity (PAGODA) study between 2002 and 2010. These subjects attended the Augusta University (AU) Medical Center and/or endocrinology clinics in the Augusta and metro-Atlanta areas of Georgia. The presence and year of diagnosis of diabetic peripheral neuropathy was identified by retrospective chart review. The vast majority of subjects were diagnosed with DPN based on a neurological history and exam by the treating endocrinologist. Many, but not all, had further evaluations and confirmation of DPN by a neurologist. Patients with diabetic foot ulcers and amputations were excluded from analysis. Patients with non-DPN microvascular complications were excluded from the control nDPN group. Medical history, clinical, and demographic profiles for T1D subjects were obtained from the medical charts (**Table 1**). All study participants gave written informed consent before enrolling in the study. The study was carried out according to The Code of Ethics of the World Medical Association (Declaration of Helsinki, 1997) and was approved by the institutional review board at AU.

**Table 1:** Demographic and Clinical variable for 694 Caucasian T1D patients without (nDPN) and with Peripheral Neuropathy (DPN).

Characteristics	nDPN (n = 507)	DPN (n = 187)	p
Gender (n)			
Males	240	77	
Females	267	110	0.174^‡^
Age (years)	39.5 ± 12.5	52.1 ± 11.5	6.5x10^-30^
Median Age (range, years)	38.8 (20.0 -73.7)	53.1 (20.5-74.5)	1.4x10^-27†^
Median Age of diagnosis (range, years)	19.0 (0-62.8)	20.1 (0-67.4)	0.372^†^
Duration of T1D (years)	17.9 ± 10.9	29.3 ± 13.0	1.2x10^-22^
UACR (ug/mg)	6.7 (1.2-2425.2)	13.1 (1.7-38205.6)	1.2x10^-8†^
BUN (mg/dL)	13.0 ± 3.8	17.1 ± 7.3	4.1x10^-10^
HbA1c (%, NGSP)	7.7 ± 0.6	8.1 ± 0.6	2x10^-3^
HbA1c (%, median (range))	7.6 (3.2 - 15.5)	7.9 (5.4 - 16.7)	0.0013^†^
HbA1c (mmol/mol, IFCC)	61.3 ± 5.7	65.5 ± 6.5	3x10^-3^
HbA1c (mmol/mol, median(range))	59.6 (31.2 - 144.5)	62.5 (38.8 - 159.0)	0.002^†^
Total Cholesterol (mg/dL)	174.8 ± 34.6	176.5 ± 39.7	0.645
LDL (mg/dL)	98.0 ± 27.7	95.7 ± 33.2	0.457
Triglycerides	91.3 ± 73.7	104.1 ± 74.1	0.07
HDL	58.8 ± 17.2	59.4 ± 18.3	0.72
Blood Pressure (mmHg)			
Systolic BP	119.2 ± 10.5	125.1 ± 11.7	4.5x10^-8^
Diastolic BP	73.5 ± 6.6	73.1 ± 7.7	0.6
MAP	88.2 ± 8.31	90.3 ± 7.8	0.005
DPN duration (years)		8.9 ± 8.5	
Other complications			
Nephropathy (n)	No	37	
Retinopathy (n)	No	100	
Hypertension (n)	74	90	
Dyslipidemia (n)	137	98	
CAD (n)	No	50	
Diabetic Foot Ulcer (n)	No	27	

CAD, Coronary artery disease.

^†^Kruskall-Wallis test, ^‡^Chi-sq Test, all other p values are from T-tests.

Venous blood was collected in clot activator tubes, allowed to clot at room temperature for 30 minutes prior to centrifugation at 3000g. Separated serum was then aliquoted into wells of 96-well plate to create a master plate. Individual daughter plates were then created by aliquoting 5-10µl of serum from this master plate. All master and daughter plates were stored at -80°C until use.

### Laboratory Measurements

We measured twenty-two proteins (IL1Ra, IL8, MCP-1, MIP-1β, CRP, SAA, MMP1, MMP2, MMP9, sgp130, sICAM1, sVCAM1, sIL2Rα, sIL6R, sTNFRI, sTNFRII, sEGFR, IGFBP1, IGFBP2, IGFBP3, IGFBP6 and tPAI1) in the serum of subjects recruited in the Phenome and Genome of Diabetes Autoimmunity (PAGODA) Study. These proteins have all been reported in the inflammation pathways associated with progression of T1D and its complications (24–26, 28, 29). Luminex assays for these proteins were obtained from Millipore (Millipore Inc., Billerica, MA, USA). Prior to the full-scale study, we optimized the dilution of sera for a distribution of intensity in the linear portion of the standard curve. Multiplex assays were performed according to the instructions provided with the kit. Briefly, serum samples were incubated with antibody coated microspheres, followed by biotinylated detection antibody. Detection of the proteins was accomplished by incubation with phycoerythrin-labeled streptavidin. The resultant bead immuno-complexes were then read on a FLEXMAP3D (Luminex, TX, USA) with the instrument settings recommended by manufacturer.

The captured median fluorescence intensity (MFI) data was passed through quality control steps as described in our earlier study (30). Briefly, wells with individual bead counts below 30, or bead count coefficient of variation (CV) above 200 were flagged for exclusion. Replicate wells with CV≥25% were excluded from further analyses. The standard concentration and MFI were log2 transformed prior to regression. Protein concentrations were estimated using a regression fit to the standard curve with serial dilution of known concentration for each protein (30).

### Construction of Inflammation Score

Ridge regression (R package “glmnet”) was used to create a multi-protein score that accounted for serum levels of multiple proteins (31). The algorithm combines the serum levels of multiple proteins in a linear combination by using the sum of the squares of the coefficients of individual proteins generated in the model based on a penalty term. The effect of the penalty term is adjusted by providing a matrix of values for lambda from 0 to infinity. The optimum lambda was determined using the lambda.min function in R, which chooses the minimum lambda value on cross validation. We optimized the model by using recursive feature elimination. We fit all 22 proteins to the peripheral neuropathy outcome using a least absolute shrinkage and selection operator (LASSO) by setting alpha=0 in the cv.glmnet function. We manually removed the proteins contributing the lowest weight to the linear predictor (**Supplementary Figure 2**). After the selection of proteins for the final model, we ran ridge regression using cv.glmnet function to obtain a linear predictor. We used the composite score obtained from the ridge regression model to calculate the odds ratios (OR) of having DPN for the upper four quintiles using the first quintile as reference.

### Statistical Analysis

Data is presented as count, percentages, and means ± standard deviation for normally distributed variables. Median and range is presented for non-normal variables. Prior to any statistical analysis, protein concentration data was log2 transformed to follow the normal distribution. For generation of the figures, the data was back transformed to natural units. For all statistical analysis we removed T1D subjects with diabetic foot ulcer (n=27). Potential univariate differences between T1D patients without any complication (nDPN) and T1D patients with diabetic neuropathy (DPN) was examined using boxplots and t-test. Pairwise correlations between individual proteins were determined using Pearson correlation coefficient, and presented as a heatmap with hierarchical clustering. The association between the serum levels of each candidate molecule and age and T1D duration was determined using a linear regression including sex and disease status as covariates.

For all association analysis, log2 transformed concentration data was converted into z-scores by centering and scaling to unit standard deviation. The potential relationship between DPN and inflammatory proteins was evaluated using logistic regression models where presence/absence of DPN was incorporated as dependent variable. Covariates, Age, sex, duration of T1D, HbA1c, hypertension (HTN), and dyslipidemia were adjusted in separate models in the logistic regression.

We divided serum level for each protein into 5 quintiles containing 20% DPN patients in each quintile (20^th^ percentile). The cutoff protein levels from DPN patients were then used to count nDPN and DPN subjects in each quintile. The 1st quintile was used as reference and OR for DPN was calculated for the upper four quintiles. Pearson’s Chi-squared test with Yates’ continuity correction was used to calculate the ORs. The chi-squared test for trend in proportions was used to calculate the p-value of overall trend.

All P-values were two-tailed and a P< 0.05 was considered statistically significant. All statistical analyses were performed using the R language and environment for statistical computing (v 4.0.3; R Foundation for Statistical Computing; www.r-project.org).

## Results

### Description of the Study Participants

Serum samples from T1D subjects (n=694) were analyzed in this study. These subjects were divided into, T1D with no complications (nDPN, n=507) and T1D patients who were diagnosed with peripheral neuropathy (DPN, n=187). The patients in DPN group were older (median age: 53.1 vs 38.8 years) and had type-1 diabetes for a longer duration (29.3 ± 13.0 vs 17.9 ± 10.9 years) than the nDPN group (**Table 1**). Subjects in DPN group have higher values for HbA1c and systolic blood pressure. There were no differences in the serum level of HDL, LDL, or cholesterol between the DPN and nDPN groups (**Table 1**). Of the 187 T1D subjects with DPN, 37 had diabetic nephropathy, 100 had retinopathy, 50 had coronary artery disease, and 27 had diabetic foot ulcer(s). Hypertension and dyslipidemia were present in both the groups (**Table 1**).

### Relationships of Inflammatory Proteins in DPN Patients

**Figure 1:**
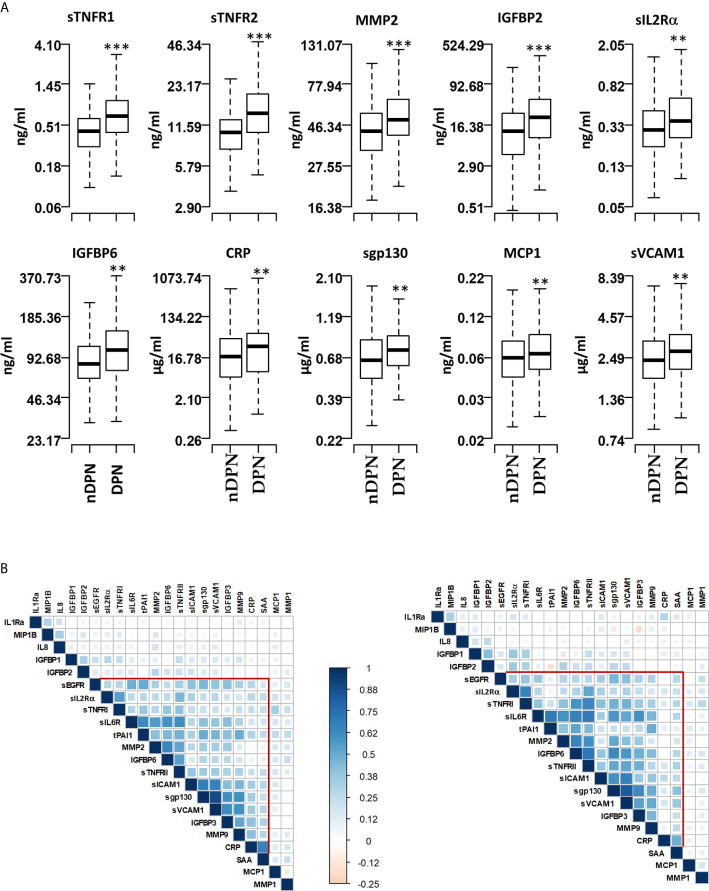
Serum levels of proteins and pairwise correlations. **(A)** Boxplots showing the measured levels of selected proteins in T1D patients without diabetic neuropathy (nDPN, n=507) and with diabetic neuropathy (DPN, n=187). **(B)** Heatmaps showing the pairwise correlation coefficients in nDPN (left) and DPN (right) patients. The correlation coefficients are clustered based on the supervised hierarchical clustering. The color density represents the strength of correlation (r) values and the size of the squares represent the significance. ***P < 0.000001, **P < 0.01.

We evaluated the serum level of 22 inflammatory proteins which have been associated with T1D progression and complications (24–26, 29). Six out of twenty-two proteins (sTNFRI, sTNFRII, MMP2, IGFBP2, sIL2Rα, IGFBP6, CRP) showed elevated (1.2-1.8 fold) mean serum levels in the DPN group compared to the nDPN group (**Figure 1** and **Supplementary Figure 1**). There was a slight but significant elevation in serum levels of sgp130 (1.16-fold), sICAM1 (1.13-fold), MCP1 (1.14-fold) and sVCAM1 (1.16-fold). Six proteins, MMP1, IGFBP1, SAA, IL1Ra, sEGFR, and IL8 showed non-significant elevation in DPN group (**Figure 1** and **Supplementary Figure 1**).

We next evaluated the correlation structure of these inflammatory proteins by pairwise Pearson’s correlation analysis in the nDPN and DPN groups separately. Hierarchical clustering of the correlation matrix suggests a single cluster with 11 proteins in the T1D group. Within this cluster, the first sub-cluster, including proteins sIL6R, tPAI1, MMP2, IGFBP6 and sTNFRII, had correlation coefficient (r) values ranging from 0.41 to 0.65 (p<10^-32^). The second sub-cluster of highly correlated group of proteins contained sICAM1, sgp130, sVCAM1, and IGFBP3 (r=0.42-0.90, p<10^-32^, **Figure 1B**, left). In the DPN group, we observed a stronger correlation among the 11 proteins compared to the nDPN group (**Figure 1B**, right). The first sub-cluster had r-values in range of 0.41-0.7 (p=0-4x10^-8^), the second cluster had r-values in range of 0.38-0.83 (p=0-8x10^-7^). The most notable difference was the strength of correlation among sEGFR, sIL2Rα, sTNFRI, sIL6R, tPAI1, MMP2, IGFBP6 and sTNFRII in the DPN group. These results suggest that inflammation plays a more coordinated role in patients with DPN compared to nDPN subjects.

### Influence of Age, Duration of Diabetes and Gender on Serum Levels

In nDPN group, the subject’s age at the time of sample collection was significantly correlated with serum levels of IGFBP2 (r=0.27, p=1x10^-9^), MMP1 (r=0.161, p=0.0003), and SAA (r=0.12, p=0.009) (**Supplementary Table 1**). The subject’s duration of T1D disease burden was significantly correlated with IGFBP1 (r=0.16, p=0.0003), IGFBP2 (r=0.26, p=2.2x10^-9^), MMP2 (r=0.22, p=1x10^-6^), and sTNFR2 (r=0.18, p=7.2x10^-5^). Females have a higher serum level on average than males for IL-1Ra (F/M ratio=1.48, p=2.8x10^-5^), IGFBP1 (F/M ratio=17.6, p=1.2x10^-8^), IGFBP2 (F/M ratio=156, p=1.2x10^-3^), IGFBP6 (F/M ratio=0.76, p=2.4x10^-7^).

In DPN group, the subject’s age at the time of sample collection was significantly correlated with serum levels of IGFBP3 (r=-0.2, p=0.008) and MMP2 (r=0.2, p=0.007). Only MMP2 (r=0.19, p=0.008) showed significant correlation with disease duration in DPN group. Only IGFBP6 (F/M ratio=0.74, p=0.007) showed significant differences between females and males in the DPN group.

We also evaluated the relationship between hemoglobin A1c (HbA1c) and serum levels of inflammatory proteins. A positive correlation was observed for CRP in the nDPN group (r=0.18, p=0.0002, **Supplementary Table 4**). A negative correlation was observed for MIP1β with HbA1c in DPN group (r=-0.3, p=0.001, **Supplementary Table 4**). Only serum concentration of MCP1 was correlated with systolic blood pressure (r=0.2, p=3.7x10^-5^)

### DPN Is Associated With Increase in Serum Levels of Inflammatory Proteins

We assessed the difference in protein level between the DPN and nDPN groups adjusting for clinical confounders in a multivariate logistic regression model (**Table 2**). The crude OR (95% CI) for the presence of DPN was significant for per SD increase in protein concentration for 15 proteins (Model 1, **Table 2**). The positive associations remained significant after multivariate adjusting of age, duration of diabetes and gender (Model 2, **Table 2**), glycemic control (Model 3, **Table 2**), hypertension (HTN) and dyslipidemia (Model 4, **Table 2**). The associations remained unchanged for sTNFR1, sIL2Rα, sTNFRII, IGFBP6 and CRP (**Table 2**). We did not observe any weakening of the associations for these five proteins in any of the models presented in **Table 2**.

**Table 2:** DPN is independently associated with per SD increase in serum levels, after adjusting for age at sample, duration of T1D, Gender, glycemic control, hypertension and dyslipidemia.

Proteins	Model 1 OR (95% CI)	Model 2 OR (95% CI)	Model 3 OR (95% CI)	Model 4 OR (95% CI)
sTNFRI	2.12(1.71-2.67)^¶^	1.82(1.45-2.32)^¶^	1.76(1.37-2.3)^‡^	1.72(1.36-2.19)^‡^
sIL2Rα	1.48(1.23-1.78)^†^	1.36(1.11-1.68)^†^	1.39(1.11-1.75)^†^	1.4(1.13-1.73)^†^
sTNFRII	2(1.53-2.65)^¶^	1.47(1.17-1.91)^†^	1.39(1.1-1.81)^†^	1.45(1.16-1.9)^†^
IGFBP6	1.75(1.38-2.24)^¶^	1.56(1.21-2.06)^†^	1.53(1.17-2.07)^†^	1.51(1.17-2.01)^†^
CRP	1.53(1.23-1.92)^†^	1.47(1.15-1.91)^†^	1.45(1.1-1.92)^†^	1.47(1.13-1.92)^†^
IGFBP1	1.32(1.09-1.6)^†^	1.13(0.91-1.43)	1.14(0.9-1.47)	1.2(0.94-1.52)
sgp130	1.32(1.06-1.68)^*^	1.25(0.98-1.61)	1.2(0.93-1.58)	1.21(0.95-1.59)
IL1Ra	1.53(1.16-2.03)^†^	1.32(0.95-1.83)	1.25(0.89-1.77)	1.24(0.89-1.73)
SAA	1.37(1.1-1.73)^†^	1.23(0.95-1.6)	1.25(0.94-1.67)	1.18(0.91-1.55)
IGFBP2	1.82(1.41-2.36)^¶^	1.14(0.87-1.51)	1.16(0.85-1.58)	1.2(0.9-1.61)
sICAM1	1.24(1.03-1.5)^*^	1.16(0.94-1.44)	1.08(0.86-1.37)	1.14(0.92-1.42)
sVCAM1	1.25(1.03-1.55)^*^	1.16(0.93-1.47)	1.12(0.88-1.45)	1.15(0.91-1.47)
MMP2	1.56(1.24-1.99)^†^	1.1(0.88-1.39)	1.1(0.86-1.42)	1.12(0.89-1.42)
MCP1	1.36(1.12-1.66)^†^	1.14(0.91-1.42)	1.09(0.86-1.4)	1.1(0.88-1.38)
MMP1	1.26(1.03-1.54)^*^	1.16(0.92-1.47)	1.11(0.86-1.44)	1.08(0.85-1.38)
IGFBP3	0.89(0.74-1.06)	0.97(0.79-1.23)	0.96(0.76-1.25)	0.94(0.77-1.2)
MIP1B	1.22(0.92-1.62)	1.2(0.87-1.65)	1.43(0.99-2.07)	1.21(0.87-1.68)
MMP9	1.05(0.87-1.28)	1.18(0.94-1.5)	1.09(0.86-1.41)	1.14(0.91-1.46)
sIL6R	1.09(0.91-1.34)	1.13(0.91-1.43)	1.06(0.84-1.34)	1.15(0.92-1.46)
sEGFR	0.86(0.71-1.02)	0.85(0.7-1.05)	0.8(0.64-1.01)	0.84(0.69-1.03)
tPAI1	0.96(0.81-1.16)	1.07(0.86-1.36)	1.02(0.8-1.3)	1.04(0.83-1.32)
IL8	1.15(0.95-1.39)	0.99(0.79-1.24)	1.01(0.79-1.28)	1.01(0.8-1.27)

Values presented are Odds ratio (OR, 95% CI). Model 1: protein concentration only, Model 2 = protein concentration + age+ duration of T1D + gender, Model 3= Model 2+ HbA1c, Model 4 = Model 2 + hypertension and dyslipidemia. *P < 0.05, ^†^P < 0.01, ^‡^P < 0.001, and ^¶^P < 1x10^-4^.

**Figure 2:**
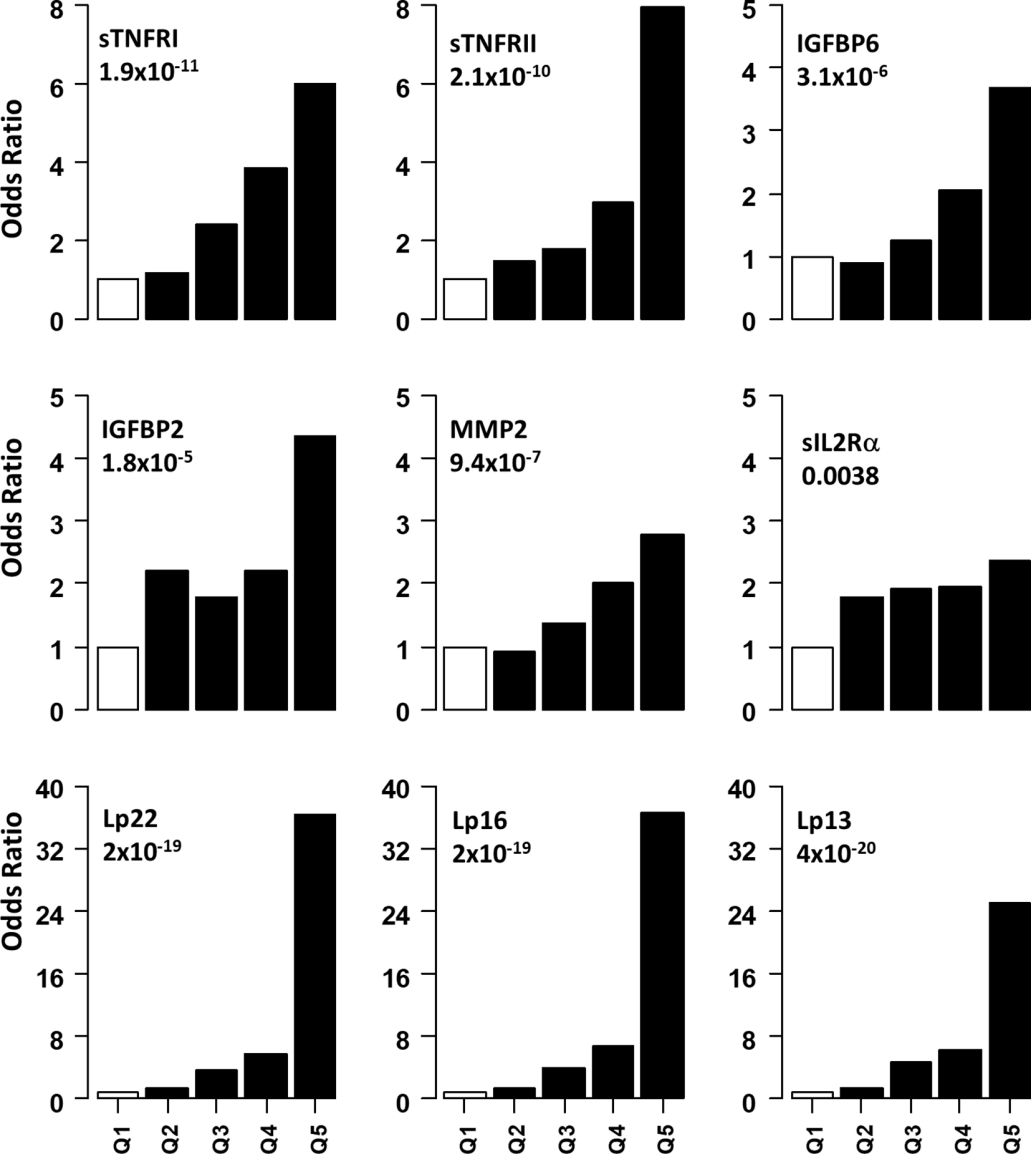
Association of serum level of proteins with presence of DPN. Odds ratios associated with each of the top four quintiles compared to the bottom 1^st^ quintile for each of the twelve individual proteins. The open bar represents the 1^st^ quintile as reference (OR = 1). From left to right, each of the other four solid bars represents the 2^nd^ to 5^th^ quintile (20% of the DPN patients). Odds ratios associated with the risk scores calculated based on different combinations of 22 (Lp22), 16, (Lp), 13(Lp) and 11 (Lp) proteins using ridge regression. Vertical axes are odds ratios.

Non-linear associations are difficult to detect on the logistic regression models we described. Thus, we determined odds ratio (OR) after dividing the protein concentrations into quintiles (**Figure 2** and **Table 3**). The serum levels of each protein in the DPN group were divided into quantiles containing 20 percent of subjects. The cutoff values obtained from the DPN patients was used to assign the T1D patients into five quantiles. For all comparisons, we used the first quintile as a reference to calculate the OR for quintiles 2-5. **Figure 2** and **Table 3** shows the odds ratio with 95% CI. We observed that DPN is associated with an increase in serum levels of 15 of the 22 proteins measured. The strongest association was noted for sTNFRI (p<1.5x10^-15^), followed by sTNFRII (p<1.5x10^-15^), IGFBP6 (p<7.1x10^-8^), IGFBP2 (p<5.7x10^-6^) and MMP2 (p<9.4x10^-5^). The OR for these proteins increased with the serum levels in the fifth quintile to 11.2 (sTNFRI), 8.69 (sTNFRII), 4.8 (IGFBP6) and 3.13 (IGFBP2). Results on MMP2 levels suggested that any increase over 20^th^ percentile has an OR of 2-4.5 of having DPN in these individuals. Markers of endothelial dysfunction (sgp130, sICAM1, sVCAM1) showed an increase in OR of from 1.5 to 2.5 as the levels of these proteins increased in the serum (**Supplementary Figure 3**).

**Table 3:** Odds ratio (95% CI) of having DPN based on serum levels in quintile (20^th^ percentile) categories.

Protein	Quintile-2 OR	Quintile -3 OR	Quintile-4 OR	Quintile-5 OR	Adj P Trend
sTNFRI	1.35 (0.82 - 2.23)	2.21 (1.32 - 3.71)	4.12 (2.37 - 7.15)	11.19 (5.75 - 21.77)	1.5x10^-16^
sTNFRII	1.32 (0.8 - 2.17)	3.03 (1.78 - 5.16)	4.3 (2.47 - 7.49)	8.69 (4.64 - 16.28)	1.5x10^-16^
IGFBP6	1.03 (0.62 - 1.7)	1.51 (0.9 - 2.53)	2.49 (1.45 - 4.27)	4.79 (2.65 - 8.67)	7.1x10^-9^
IGFBP2	0.91 (0.55 - 1.5)	1.5 (0.89 - 2.52)	2.38 (1.38 - 4.09)	3.13 (1.79 - 5.48)	5.7x10^-7^
MMP2	1.99 (1.19 - 3.33)	2.16 (1.29 - 3.63)	2.11 (1.26 - 3.54)	4.52 (2.57 - 7.95)	9.4x10^-7^
sIL2Rα	1.69 (1.01 - 2.83)	1.72 (1.03 - 2.88)	1.78 (1.06 - 2.98)	3.07 (1.78 - 5.3)	0.00030
sgp130	1.53 (0.89 - 2.63)	2.29 (1.31 - 4)	2.32 (1.33 - 4.06)	2.25 (1.29 - 3.93)	0.00088
sVCAM1	1.02 (0.6 - 1.74)	1.42 (0.82 - 2.46)	2.57 (1.43 - 4.61)	1.6 (0.92 - 2.79)	0.00620
SAA	1.61 (0.93 - 2.78)	1.7 (0.98 - 2.95)	1.89 (1.09 - 3.29)	2.19 (1.25 - 3.84)	0.00640
MCP1	1.42 (0.83 - 2.42)	1.64 (0.96 - 2.81)	1.16 (0.69 - 1.96)	2.76 (1.56 - 4.87)	0.0130
CRP	1.39 (0.8 - 2.41)	1.08 (0.63 - 1.86)	2 (1.13 - 3.53)	1.94 (1.1 - 3.41)	0.0130
sICAM1	1.58 (0.91 - 2.73)	2.04 (1.17 - 3.57)	2.04 (1.17 - 3.57)	1.83 (1.05 - 3.18)	0.0130
MMP1	1.46 (0.87 - 2.46)	1.29 (0.77 - 2.16)	1.56 (0.92 - 2.63)	1.95 (1.14 - 3.33)	0.0200
IGFBP1	1.01 (0.61 - 1.68)	1.53 (0.9 - 2.59)	1.39 (0.82 - 2.35)	1.63 (0.96 - 2.77)	0.0240
IL1Ra	1.31 (0.77 - 2.24)	1.51 (0.88 - 2.6)	1.33 (0.78 - 2.28)	1.94 (1.12 - 3.37)	0.0340
sIL6R	1.51 (0.9 - 2.54)	1.71 (1.01 - 2.9)	1.52 (0.9 - 2.56)	1.67 (0.99 - 2.83)	0.057
IGFBP3	0.93 (0.52 - 1.67)	0.51 (0.29 - 0.9)	0.71 (0.4 - 1.26)	0.61 (0.34 - 1.08)	0.066
sEGFR	1.6 (0.91 - 2.8)	1 (0.59 - 1.71)	0.86 (0.51 - 1.46)	0.78 (0.46 - 1.32)	0.066
MIP1β	1.08 (0.63 - 1.84)	1.3 (0.76 - 2.23)	1.19 (0.7 - 2.03)	1.54 (0.89 - 2.66)	0.110
IL8	1.05 (0.6 - 1.83)	1.15 (0.66 - 2.02)	1.44 (0.81 - 2.55)	1.28 (0.73 - 2.25)	0.180
MMP9	0.98 (0.56 - 1.7)	1.05 (0.6 - 1.84)	0.96 (0.55 - 1.67)	1.15 (0.66 - 2.02)	0.660
tPAI1	1.24 (0.71 - 2.18)	0.94 (0.54 - 1.63)	1.06 (0.61 - 1.85)	1 (0.58 - 1.74)	0.720
Lp22	1.51 (0.82 - 2.79)	3.76 (1.95 - 7.25)	5.74 (2.88 - 11.46)	36.34 (12.17 - 108.5)	1.9x10^-19^
Lp16	1.45 (0.79 - 2.68)	3.96 (2.05 - 7.66)	6.71 (3.31 - 13.6)	36.56 (12.25 - 109.15)	1.9x10^-20^
Lp13	1.38 (0.75 - 2.55)	4.74 (2.42 - 9.3)	6.41 (3.18 - 12.94)	25.16 (9.57 - 66.13)	4x10^-20^
Lp11	1.42 (0.77 - 2.62)	4.19 (2.16 - 8.13)	8.94 (4.26 - 18.77)	19.47 (7.98 - 47.47)	1.9x10^-20^

Quintile 1 was used as a reference. Lp: linear predictor derived from combination of 22 (Lp22), 16 (Lp16) and 13 (Lp13) proteins.

We implemented a machine-learning approach using ridge regression to find the a combination of proteins optimally associated with DPN. The combination of 22 proteins gave the highest OR of 36.3 in 5^th^ quintile, which was higher than that of any single protein. We then proceeded to determine the minimum number of proteins that can be combined together to improve the OR of having DPN. We found an optimal combination of 16 molecules (IL1Ra, MCP1, MIP1β, CRP, IGFBP2, IGFBP3, IGFBP6, MMP1, MMP2, MMP9, sEGFR, sgp130, sIL2Rα, sTNFRI, sTNFRII and tPAI1) which still have comparable ORs in the 3^rd^-5^th^ quintile (**Figure 2** and **Table 3**). Our composite score results suggest three different risk groups in DPN patients, group 1 includes low risk DPN patients who have serum levels in 2^nd^ quintile (OR<1.5). The individuals in 3^rd^ and 4^th^ quintile represents medium risk group of DPN patients with OR between 3.5-9.0. The individuals in the highest quintile belongs to the highest risk and depending on the combination of protein may have ORs between 20-36.

## Discussion

We report increased serum levels of five out of twenty-two proteins assessed in T1D patients with DPN. The differences were significant after accounting for age, duration of diabetes, and gender. The strongest associations with DPN were with the two TNF-α receptors (TNFR1 and TNFR2). Moderate associations were observed for solubleIL2 receptor (sIL2Rα), a marker of activated helper T-cell marker, as well as IGF binding protein (IGFBP6) and, CRP, a marker of inflammation. Using a quintile-based risk assessment approach, we identified several proteins associated with increased risk of DPN in T1D patients. Increase in serum levels of SAA, MMP2, sIL2Rα, sIL1Ra, sgp130, sICAM-1 and sIL6R in the 2^nd^-5^th^ quintiles were significantly associated with DPN, whereas serum levels in 3^rd^ to 5^th^ quintile showed associations with DPN for IGFBP1, IGFBP2, IGFBP6 and sVCAM1. The associations observed in this study are potential therapeutic targets for DPN prevention. The TNF-α/IL-6 pathway is reportedly involved in DPN pathogenesis (20, 26).

The higher levels of inflammation markers in the DPN group supports association between inflammation and pathogenesis of DPN. Our results on the serum level of sTNFRI and sTNFRII are in agreement with previous report showing activation of TNF-α system in T1D patients with nephropathy, retinopathy and neuropathy (23–27). The soluble form of TNFRs are known to act as stabilizers of TNF-α activity, which prolongs systemic and vascular inflammation associated with complications (32). We did not noticed any weakening of the associations with DPN for sTNFRII for any of the models (27).

Activation of the TNF-α system is associated with activation of T-cells (33), which is evident with increase in serum levels of sIL2Rα, a marker for activated T-cells in several autoimmune diseases including T1D. We have not identified any other study that has evaluated the association between sIL2Rα and presence of DPN in T1D patients. Contrary to a previously published report (27) CRP was strongly associated with presence of DPN in our study. This indicates role of systemic inflammation in DPN. In our study, markers of endothelial dysfunctions (IL6, sgp130, sVCAM1, sICAM1) were not significant after multivariate adjustment. We measured serum levels of two proteins that are required for IL6 signaling, sgp130 and sIL6R. Classical IL6 signaling is only limited to immune cells. The majority of the cell types’ action of IL6 is dependent on its binding to soluble IL6 receptor (sIL6R) and a signal transducing glycoprotein, sgp130, on the cell *via* trans-signaling (34). In our logistic regression analysis, we did not see any significant association of these two proteins with the presence of DPN. However, our quintile analysis suggests that any increase above the 20th percentile in the serum levels for sIL6R and sgp130 is strongly associated with presence of DPN (OR=1.5-2.25, **Table 3**). The results of our study, suggests there is increase in the inflammation promoting IL6 trans-signaling in DPN patients (35). Taken together, out results suggests an activation of TNF-α/IL6 inflammatory pathways in DPN, similar to those reported in retinopathy (25) and nephropathy (24).

Chronic hyperglycemia is shown to be one of the pathogenic factors in activation of inflammatory pathways. In our analysis, the association with DPN remained unchanged for sTNFRI, sTNFRII, sIL2Rα, IGFBP6 and CRP, after adjusting for HbA1c levels in study subjects, suggesting that factors other than hyperglycemia are also involved in pathogenesis of DPN (36). Similar to earlier studies we did not see any effect of HTN and dyslipidemia (26, 27, 37). The results presented here suggests that our combined model may be a useful clinical tool to identify patients at risk for developing DPN.

We applied a ridge regression model to calculate the risk of DPN in T1D individuals. Ridge regression allows for individual coefficients to model the association between each individual protein and DPN. This model allows more model flexibility compared to the composite z-score approach to predict diabetes-related microvascular complications as reported by Schram et al. (26).

Our study limitations include its cross-sectional nature and the classification of DPN only based on chart review without further clinical validation. Although age was included as a confounder, DPN patients were still 14 years older than nDPN patients on average and aging is associated with inflammatory markers (26). The study has several strengths including measurement of multiple markers in a multiplex format, which enhances the replicability of the results. We have included proteins that are involved in the activation of inflammation (sTNFRI, sTNFRII and sIL2Rα), systemic inflammation (SAA and CRP), insulin like growth factor signaling (IGFBPs) and vascular function (sIL6R, sgp130, sVCAM1 and sICAM1). Our study has a larger DPN population (n=187) and more inflammatory markers than previously reported studies (26, 27). The most important contribution is the machine learning approach to combine the information to an individualized linear predictor score.

We report here that serum levels of sTNFRI, sTNFRII, sIL2Rα, IGFBP6 and CRP are increased in DPN patients with T1D after adjusting for confounding co-variates. We have successfully shown that the serum levels can also be used for stratifying DPN patients into risk groups. Our study indicates that DPN patients have an activation of inflammatory pathways, unaffected by glycemic control and hypertension. This suggests a new therapeutic strategy to prevent DPN. Our inflammatory signature may be of clinically useful tool to screen for activated inflammation in diabetes patients.

## Data Availability Statement

The raw data supporting the conclusions of this article will be made available by the authors, without undue reservation.

## Ethics Statement

The studies involving human participants were reviewed and approved by Augusta University IRB. The patients/participants provided their written informed consent to participate in this study.

## Author Contributions

SP and J-XS were involved with conception of the project. SP and MH acquired the Luminex data. SP, LT, and PT were responsible for data analysis. WZ, KB, DH, MG, JB, RB, BB, JM, and JR contributed to clinical samples. All authors contributed to the article and approved the submitted version.

## Funding

This work was supported by grants from the National Institutes of Health (R21HD050196, R33HD050196, and 2RO1HD37800) and JDRF (1-2004-661) to J-XS. SP (2-2011-153, 10-2006-792 and 3-2004-195) and WZ (3-2009-275) were supported by Postdoctoral Fellowship and Career Development Award from JDRF. PT was supported by NIH/NIDDK fellowship (F30DK12146101A1).

## Conflict of Interest

The authors declare that the research was conducted in the absence of any commercial or financial relationships that could be construed as a potential conflict of interest.
