# Supplementary material for: Serum Levels of Inflammatory Proteins Are Associated With Peripheral Neuropathy in a Cross-Sectional Type-1 Diabetes Cohort
Source: Front Immunol. 2021 Mar 31;12:654233. doi: 10.3389/fimmu.2021.654233 (PMC8044415; doi:10.3389/fimmu.2021.654233)
Supplement: Supplementary file 1 [file DataSheet_1.pdf]

Supplementary figure legends

**Supplementary Figure 1:** Box plots of proteins showing univariate difference between T1D with no neuropathy (nDPN) and neuropathy (DPN) patients. p-values presented in the figures are derived from Student's t-test.

**Supplementary figure 2:** Ridge regression coefficients (weights) each protein used for generating a linear predictor (Lp). Proteins that contributes least were dropped in each step to generate Lp using 22 proteins (A), using 16 proteins (B), using 13 proteins (C) and 11 proteins (D).

**Supplementary figure 3:** Odds ratios associated with each of the top four quintiles compared to the bottom $1^{st}$ quintile for each of the twelve individual proteins. The open bar represents the $1^{st}$ quintile as reference (OR = 1). From left to right, each of the other four solid bars represents the $2^{nd}$ to $5^{th}$ quintile (20% of the DPN patients). Vertical axes are odds ratios.

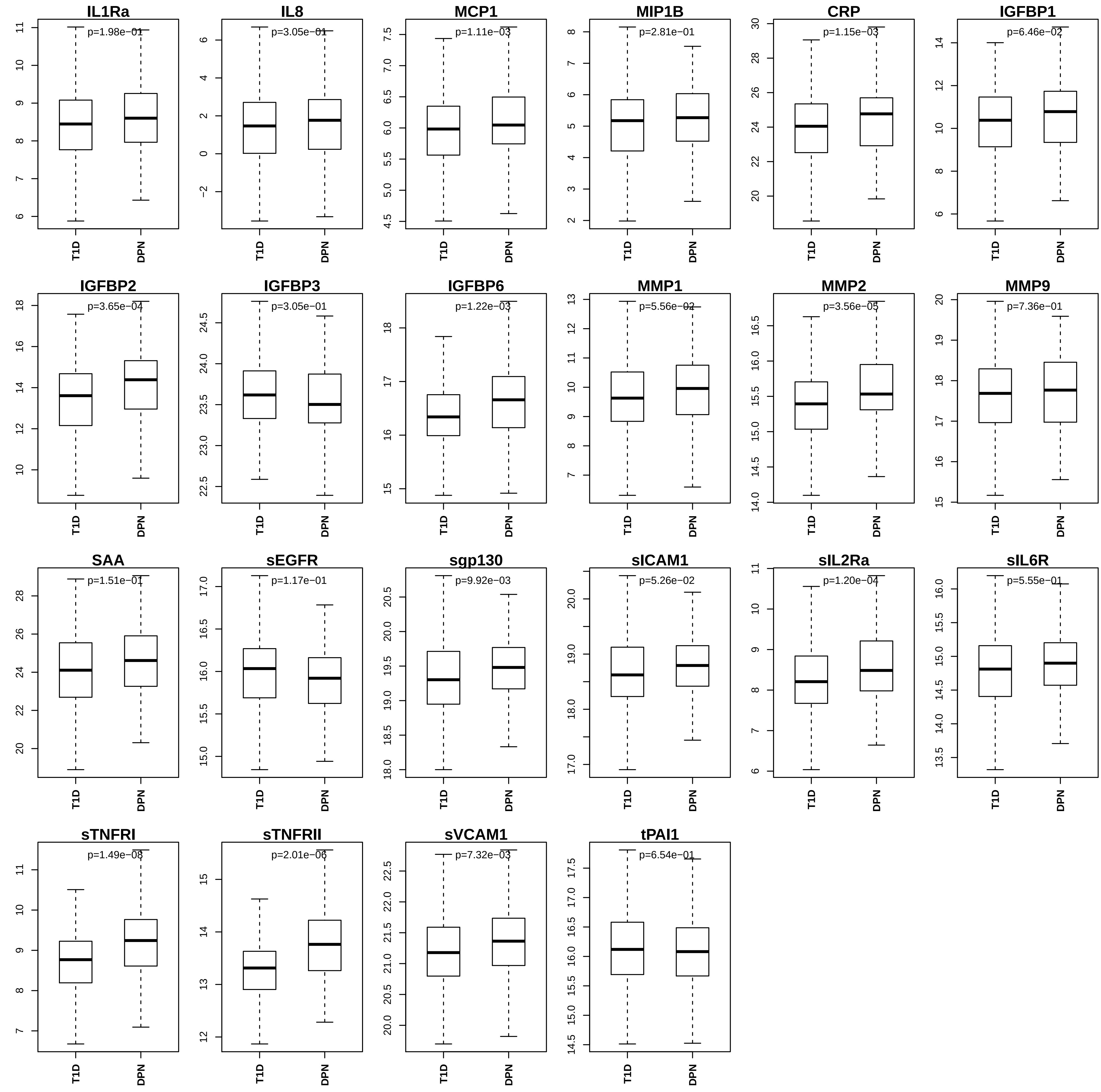
IL1Ra
p=1.98e-01
IL8
p=3.05e-01
MCP1
p=1.11e-03
MIP1B
p=2.81e-01
CRP
p=1.15e-03
IGFBP1
p=6.46e-02
IGFBP2
p=3.65e-04
IGFBP3
p=3.05e-01
IGFBP6
p=1.22e-03
MMP1
p=5.56e-02
MMP2
p=3.56e-05
MMP9
p=7.36e-01
SAA
p=1.51e-01
sEGFR
p=1.17e-01
sgp130
p=9.92e-03
sICAM1
p=5.26e-02
sIL2Ra
p=1.20e-04
sIL6R
p=5.55e-01
sTNFRI
p=1.49e-08
sTNFRII
p=2.01e-06
sVCAM1
p=7.32e-03
tPAI1
p=6.54e-01
T1D
DPN

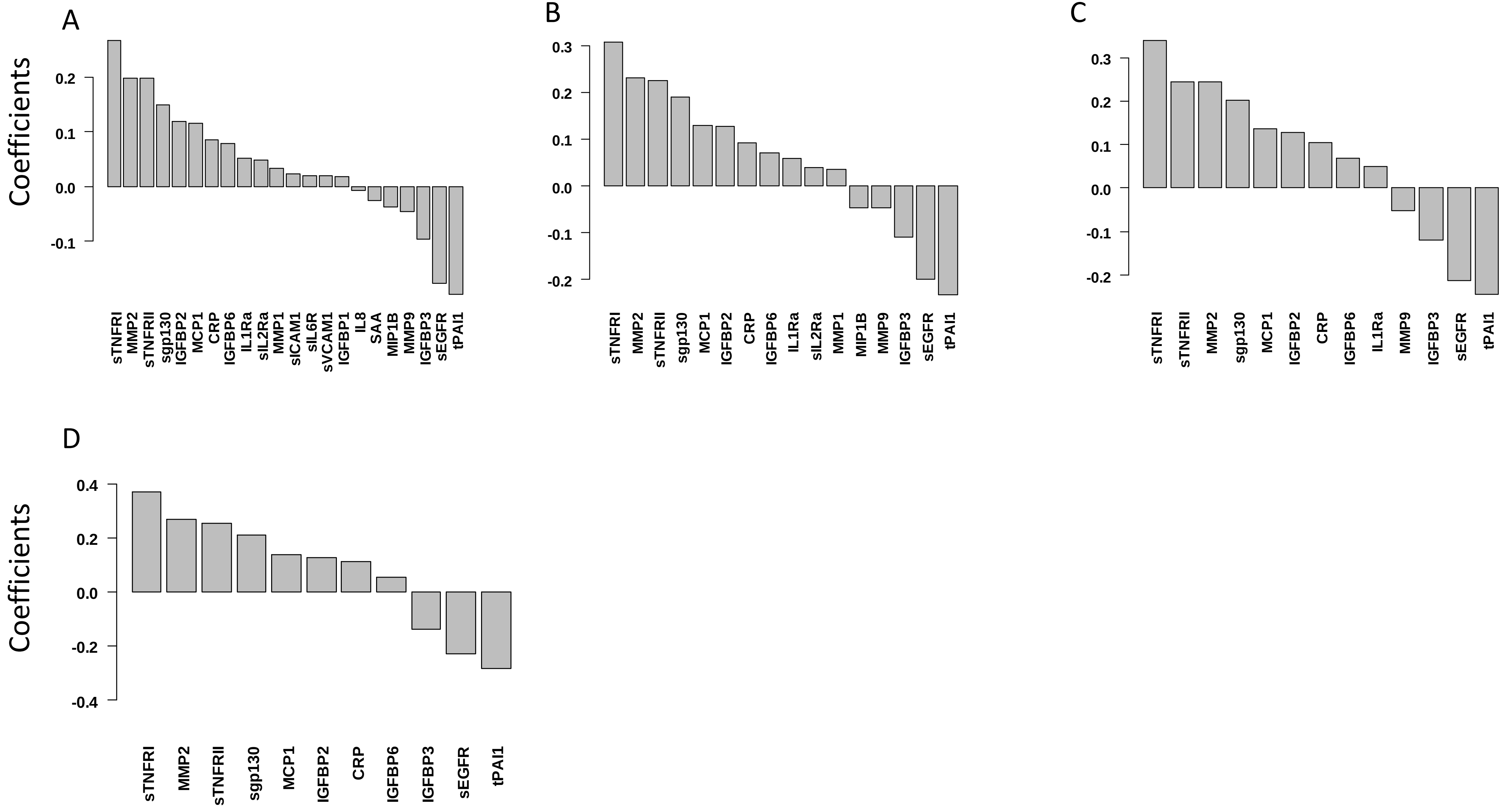


**Supplementary figure 2**

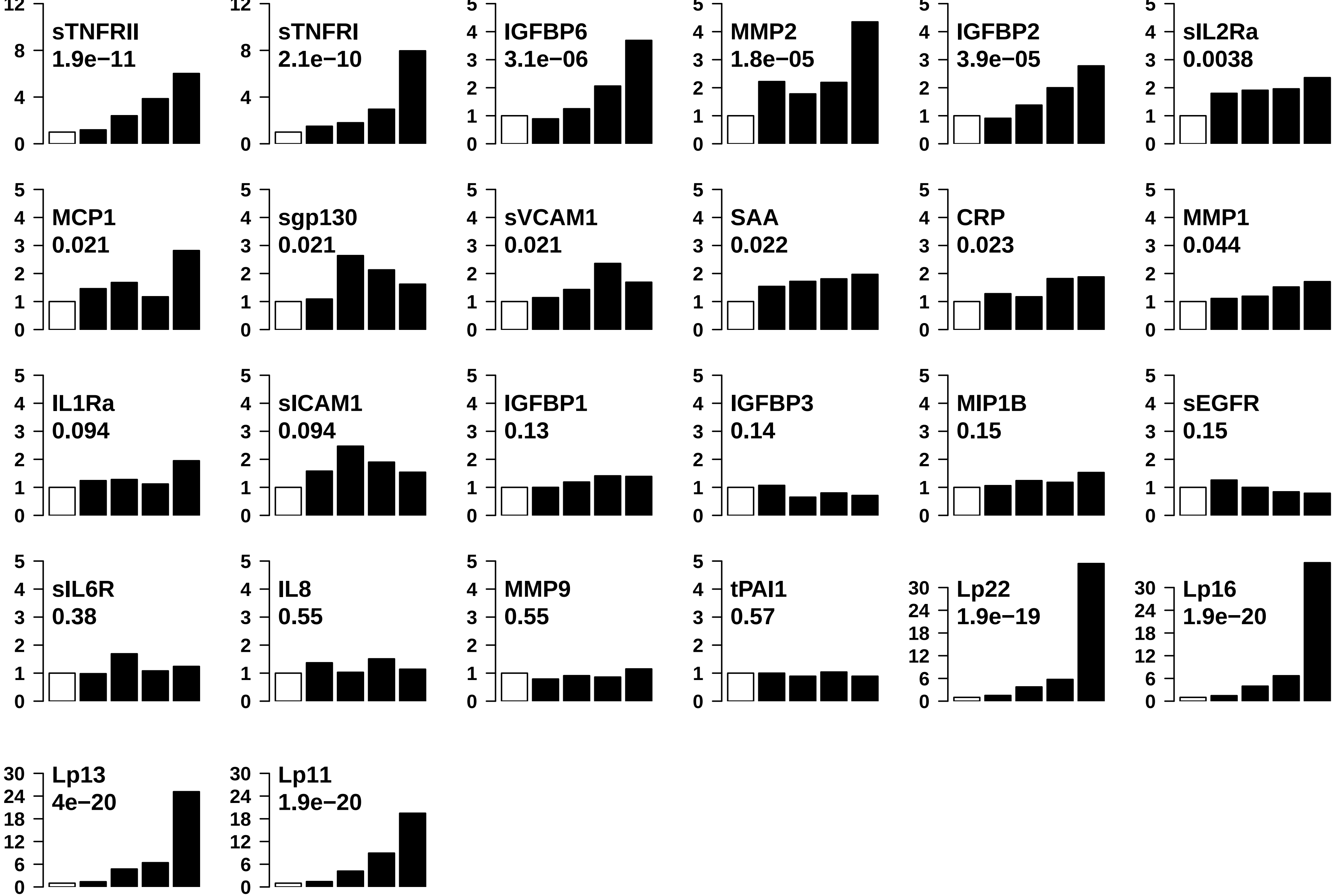
sTNFRII
1.9e−11
sTNFRI
2.1e−10
IGFBP6
3.1e−06
MMP2
1.8e−05
IGFBP2
3.9e−05
sIL2Ra
0.0038
MCP1
0.021
sgp130
0.021
sVCAM1
0.021
SAA
0.022
CRP
0.023
MMP1
0.044
IL1Ra
0.094
sICAM1
0.094
IGFBP1
0.13
IGFBP3
0.14
MIP1B
0.15
sEGFR
0.15
sIL6R
0.38
IL8
0.55
MMP9
0.55
tPAI1
0.57
Lp22
1.9e−19
Lp16
1.9e−20
Lp13
4e−20
Lp11
1.9e−20
