# Supplementary material for: Serum Levels of Inflammatory Proteins Are Associated With Peripheral Neuropathy in a Cross-Sectional Type-1 Diabetes Cohort
Source: Front Immunol. 2021 Mar 31;12:654233. doi: 10.3389/fimmu.2021.654233 (PMC8044415; doi:10.3389/fimmu.2021.654233)
Supplement: Supplementary file 2 [file DataSheet_2.docx]

**Supplementary Table 1:** Correlations between serum levels of proteins with age at which sample was drawn in non-DPN (nDPN,n=507) and DPN (n=187) group.

|  | nDPN (n=507) | | DPN (n=159) | |
| --- | --- | --- | --- | --- |
| Protein | r | p-val* | r | p-val* |
| IL1Ra | 0.10909 | 0.015381 | 0.031706 | 0.696274 |
| IL8 | 0.100785 | 0.032173 | 0.075624 | 0.381556 |
| MCP1 | 0.093427 | 0.039921 | 0.190933 | 0.01926 |
| MIP1B | 0.087147 | 0.05744 | -0.09373 | 0.255562 |
| CRP | 0.043073 | 0.357727 | 0.111281 | 0.187356 |
| IGFBP1 | 0.03092 | 0.487271 | -0.03688 | 0.644455 |
| IGFBP2 | **0.266804** | **1.04E-09** | 0.121466 | 0.12721 |
| IGFBP3 | -0.08757 | 0.061139 | **-0.22326** | **0.007356** |
| IGFBP6 | 0.067979 | 0.126351 | 0.131331 | 0.100004 |
| MMP1 | 0.160682 | 0.00028 | -0.00112 | 0.988904 |
| MMP2 | **0.178054** | **5.54E-05** | **0.172019** | **0.030679** |
| MMP9 | -0.0737 | 0.116446 | -0.16653 | 0.048427 |
| SAA | **0.122481** | **0.008692** | 0.144909 | 0.084208 |
| sEGFR | -0.03702 | 0.408315 | -0.01567 | 0.846056 |
| sgp130 | 0.01542 | 0.743442 | 0.052474 | 0.536595 |
| sICAM1 | 0.032335 | 0.492885 | 0.028414 | 0.73894 |
| sIL2Ra | 0.007558 | 0.865463 | 0.105092 | 0.187382 |
| sIL6R | 0.016819 | 0.706693 | -0.00524 | 0.948074 |
| sTNFRI | 0.071 | 0.111 | 0.126 | 0.112 |
| sTNFRII | 0.109 | 0.014333 | 0.171 | 0.032 |
| sVCAM1 | 0.044 | 0.348 | 0.080 | 0.344 |
| tPAI1 | -0.059 | 0.194 | -0.113 | 0.175 |

*p-values were not corrected for multiple comparisons

**Supplementary Table 2**: Correlations between serum levels and duration of T1D

|  | nDPN (n=507) | | DPN (n=159) | |
| --- | --- | --- | --- | --- |
| Protein | r | p-val* | r | p-val* |
| IL1Ra | 0.0925 | 0.040 | 0.0038 | 0.962 |
| IL8 | 0.0762 | 0.105683 | 0.0169 | 0.846 |
| MCP1 | 0.1112 | 0.0143 | 0.1057 | 0.198189 |
| MIP1B | 0.0635 | 0.167 | -0.1782 | 0.030 |
| CRP | 0.0238 | 0.612107 | 0.0802 | 0.343 |
| IGFBP1 | **0.1588** | **0.000331** | 0.1625 | 0.041 |
| IGFBP2 | **0.2617** | **2.19E-09** | 0.2176 | 0.006 |
| IGFBP3 | -0.0542 | 0.24671 | -0.0895 | 0.288 |
| IGFBP6 | 0.0901 | 0.042523 | 0.0203 | 0.800 |
| MMP1 | 0.0048 | 0.915 | -0.0433 | 0.590 |
| MMP2 | **0.2151** | **1.02E-06** | **0.2061** | **0.009378** |
| MMP9 | 0.0176 | 0.709 | -0.1551 | 0.066 |
| SAA | -0.0419 | 0.370 | 0.0266 | 0.753 |
| sEGFR | 0.0740 | 0.098 | 0.0385 | 0.633 |
| sgp130 | 0.0352 | 0.454 | 0.1449 | 0.087 |
| sICAM1 | 0.0515 | 0.274 | 0.1286 | 0.130 |
| sIL2Ra | 0.0779 | 0.080 | 0.1795 | 0.024 |
| sIL6R | 0.0265 | 0.553 | -0.0581 | 0.470 |
| sTNFRI | 0.0910 | 0.041 | 0.1095 | 0.169 |
| sTNFRII | **0.1760** | **7.22E-05** | 0.0457 | 0.569 |
| sVCAM1 | 0.0068 | 0.884 | 0.1524 | 0.071 |
| tPAI1 | -0.0403 | 0.379 | -0.1103 | 0.187 |

*p-values were not corrected for multiple comparisons

**Supplementary Table 3**: Gender differences in protein levels

|  | nDPN (n=507) | | DPN (n=159) | |
| --- | --- | --- | --- | --- |
| Protein | F/M | p-val* | F/M | p-val* |
| IL1Ra | **1.48** | **3.74E-07** | 1.40 | 0.839 |
| IL8 | 1.01 | 0.35757 | 1.48 | 0.982 |
| MCP1 | 0.93 | 0.19591 | 0.99 | 0.418 |
| MIP1B | 1.11 | 0.282529 | 1.35 | 0.143 |
| CRP | 1.43 | 0.00342 | 1.27 | 0.532 |
| IGFBP1 | **1.76** | **1.18E-08** | 1.41 | 0.054 |
| IGFBP2 | 1.56 | 1.17E-03 | 1.30 | 0.306 |
| IGFBP3 | 1.12 | 0.03453 | 1.04 | 0.797 |
| IGFBP6 | **0.76** | **2.94E-07** | **0.71** | **0.002** |
| MMP1 | 1.07 | 0.543 | 0.97 | 0.740 |
| MMP2 | 0.99 | 0.646 | 0.96 | 0.589 |
| MMP9 | 1.11 | 0.663 | 0.75 | 0.737 |
| SAA | 1.51 | 0.101672 | 0.90 | 0.080 |
| sEGFR | 1.00 | 0.917 | 0.94 | 0.169 |
| sgp130 | 0.98 | 0.972 | 0.93 | 0.324 |
| sICAM1 | 1.03 | 0.964 | 0.91 | 0.707 |
| sIL2Ra | 0.95 | 0.767 | 0.94 | 0.362 |
| sIL6R | 0.98 | 0.798 | 0.91 | 0.288 |
| sTNFRI | 0.89 | 0.045 | 0.82 | 0.087 |
| sTNFRII | 1.03 | 0.689 | 0.95 | 0.729 |
| sVCAM1 | 1.01 | 0.609 | 0.89 | 0.216 |
| tPAI1 | 0.93 | 0.046 | 0.85 | 0.866 |

*p-values were not corrected for multiple comparisons

**Supplementary Table 4**: Correlations with HbA1c as a measure of hyperglycemia.

|  | nDPN (n=507) | | DPN (n=159) | |
| --- | --- | --- | --- | --- |
| Protein | r | p-val* | r | p-val* |
| IL1Ra | -0.0415 | 0.392 | -0.0323 | 0.719 |
| IL8 | 0.0172 | 0.732 | 0.0081 | 0.933 |
| MCP1 | 0.0253 | 0.6046 | 0.0262 | 0.775 |
| MIP1B | -0.0641 | 0.194 | -0.2665 | 0.003 |
| CRP | 0.1841 | 0.000225 | -0.0393 | 0.674 |
| IGFBP1 | -0.0465 | 0.330 | 0.0355 | 0.690 |
| IGFBP2 | -0.0850 | 0.074 | -0.1868 | 0.034 |
| IGFBP3 | 0.0574 | 0.254 | 0.1647 | 0.075 |
| IGFBP6 | -0.0857 | 0.072 | -0.0834 | 0.349 |
| MMP1 | 0.0926 | 0.052 | 0.0306 | 0.733 |
| MMP2 | 0.0064 | 0.89 | -0.1453 | 0.102 |
| MMP9 | 0.0379 | 0.453 | 0.1403 | 0.131 |
| SAA | 0.0958 | 0.056 | 0.0316 | 0.734 |
| sEGFR | 0.1170 | 0.015 | -0.0379 | 0.672 |
| sgp130 | 0.0391 | 0.440 | 0.0468 | 0.616 |
| sICAM1 | 0.0988 | 0.051 | -0.0309 | 0.742 |
| sIL2Ra | 0.0565 | 0.238 | 0.0552 | 0.535 |
| sIL6R | 0.0374 | 0.436 | 0.0045 | 0.960 |
| sTNFRI | 0.0209 | 0.661 | 0.1466 | 0.097 |
| sTNFRII | 0.0112 | 0.82 | 0.0232 | 0.795 |
| sVCAM1 | 0.0228 | 0.652 | 0.0575 | 0.538 |
| tPAI1 | 0.0485 | 0.325 | -0.0178 | 0.849 |

*p-values were not corrected for multiple comparisons

**Supplementary Table 5**: Correlations with systolic blood pressure.

|  | nDPN (n=507) | | DPN (n=159) | |
| --- | --- | --- | --- | --- |
| Protein | r | p-val* | r | p-val* |
| IL1Ra | -0.0348 | 0.471 | 0.1414 | 0.110 |
| IL8 | 0.0772 | 0.123 | -0.1136 | 0.229 |
| MCP1 | 0.1990 | 3.75E-05 | 0.1324 | 0.141 |
| MIP1B | -0.0056 | 0.909 | 0.0654 | 0.470 |
| CRP | 0.0963 | 0.054121 | 0.1489 | 0.105 |
| IGFBP1 | -0.0588 | 0.216 | 0.0052 | 0.953 |
| IGFBP2 | -0.0753 | 0.113 | -0.0851 | 0.332 |
| IGFBP3 | 0.0187 | 0.709 | -0.0698 | 0.447 |
| IGFBP6 | -0.0149 | 0.753 | 0.0313 | 0.722 |
| MMP1 | 0.0524 | 0.270 | -0.1124 | 0.203 |
| MMP2 | 0.0678 | 0.15 | -0.0266 | 0.763 |
| MMP9 | 0.0970 | 0.053 | 0.0752 | 0.417 |
| SAA | 0.1530 | 0.002 | -0.0112 | 0.903 |
| sEGFR | 0.0263 | 0.582 | -0.0749 | 0.399 |
| sgp130 | 0.0580 | 0.249 | 0.0473 | 0.609 |
| sICAM1 | 0.0708 | 0.160 | 0.1164 | 0.209 |
| sIL2Ra | 0.0035 | 0.941 | -0.0593 | 0.500 |
| sIL6R | -0.0110 | 0.818 | -0.0391 | 0.659 |
| sTNFRI | 0.0649 | 0.172 | 0.0465 | 0.597 |
| sTNFRII | 0.0322 | 0.50 | 0.1029 | 0.244 |
| sVCAM1 | 0.0595 | 0.236 | 0.0899 | 0.331 |
| tPAI1 | 0.1085 | 0.026 | 0.0028 | 0.976 |

*p-values were not corrected for multiple comparisons
